# Navigating imaging artifacts: challenges with visualizing intrinsically fluorescent antimalarials

**DOI:** 10.3389/fpara.2026.1919904

**Published:** 2026-08-17

**Authors:** Emily K. Bremers, David Anaguano, Paul R. Carlier, Vasant Muralidharan, Maria Belen Cassera

**Affiliations:** 1 Department of Biochemistry and Molecular Biology, University of Georgia, Athens, GA, United States; 2 Center for Tropical and Emerging Global Diseases, University of Georgia, Athens, GA, United States; 3 Department of Cellular Biology, University of Georgia, Athens, GA, United States; 4 Department of Pharmaceutical Sciences, University of Illinois Chicago, Chicago, IL, United States; 5 Department of Chemistry, University of Illinois Chicago, Chicago, IL, United States

**Keywords:** antiparasitic compounds, autofluorescence, fluorescence microscopy, intrinsic fluorescent, tetrahydro-ß-carboline

## Abstract

Elucidating the target of an antiparasitic small molecule can aid in the drug development process. However, various technical challenges related to parasite biology prevent timely target identification. Microscopy methods determining the co-localization of a drug with subcellular markers is a powerful approach that provides evidence of its localization within the cell. This technique can help pinpoint the primary site of action of novel therapeutics, which can assist in elucidating their mode of action. Intrinsically fluorescent drugs allow for subcellular localization without extensive medicinal chemistry. We imaged the intrinsically fluorescent drugs PRC1590 and quinine with digestive vacuole stains to determine these compounds’ localization in the malaria parasite, *Plasmodium falciparum*. We observed that imaging intrinsically fluorescent compounds often produces imaging artifacts due to spectral overlap and autofluorescence of *P. falciparum*, resulting in false positive co-localization findings. Our work outlines critical methodological considerations when studying subcellular localization of intrinsically fluorescent antimalarials.

## 1 Introduction

Understanding the mode of action (MOA) of an antiparasitic compound can aid in the development of safe and effective drugs. Knowledge of the molecular target of a lead compound can inform medicinal chemistry efforts that reduce off-target effects and improve the efficacy of related analogs (Davis, 2020). Target-based approaches can accelerate the drug discovery process (Moffat et al., 2017), which is urgently needed for eukaryotic parasitic diseases such as malaria. However, identifying the molecular targets of antiparasitic compounds presents several challenges. Multiple cellular targets, parasite biological complexity, and host-cell contamination can all complicate and hinder target deconvolution efforts (Müller and Hemphill, 2016).

Localizing a small molecule in a parasite is one experimental approach that has been used to interrogate the MOA of a novel compound. For example, a fluorescent probe of primaquine was observed to localize to the cytosol in the asexual blood stage of the malaria parasite (McQueen et al., 2017), suggesting a cytoplasmic molecular target. However, attaching a bulky fluorophore to a small molecule of interest can require extensive medicinal chemistry (Broichhagen and Kilian, 2021), or could alter the mechanism of action of the original compound. Utilizing the intrinsic fluorescence of a drug can help circumvent these technical challenges. Aromatic compounds fluoresce due to their chemical structure that can be visualized with microscopy (Yin et al., 2020). For example, previous work on diamidines in multiple parasites have identified that this intrinsically fluorescent drug colocalizes with DNA in *P. falciparum* (Purfield et al., 2009), *Trypanosoma cruzi* (Girard et al., 2016), *Trypanosoma brucei* (Mathis et al., 2006), and *Leishmania donovani* (Yang et al., 2016).

**Figure 1:**
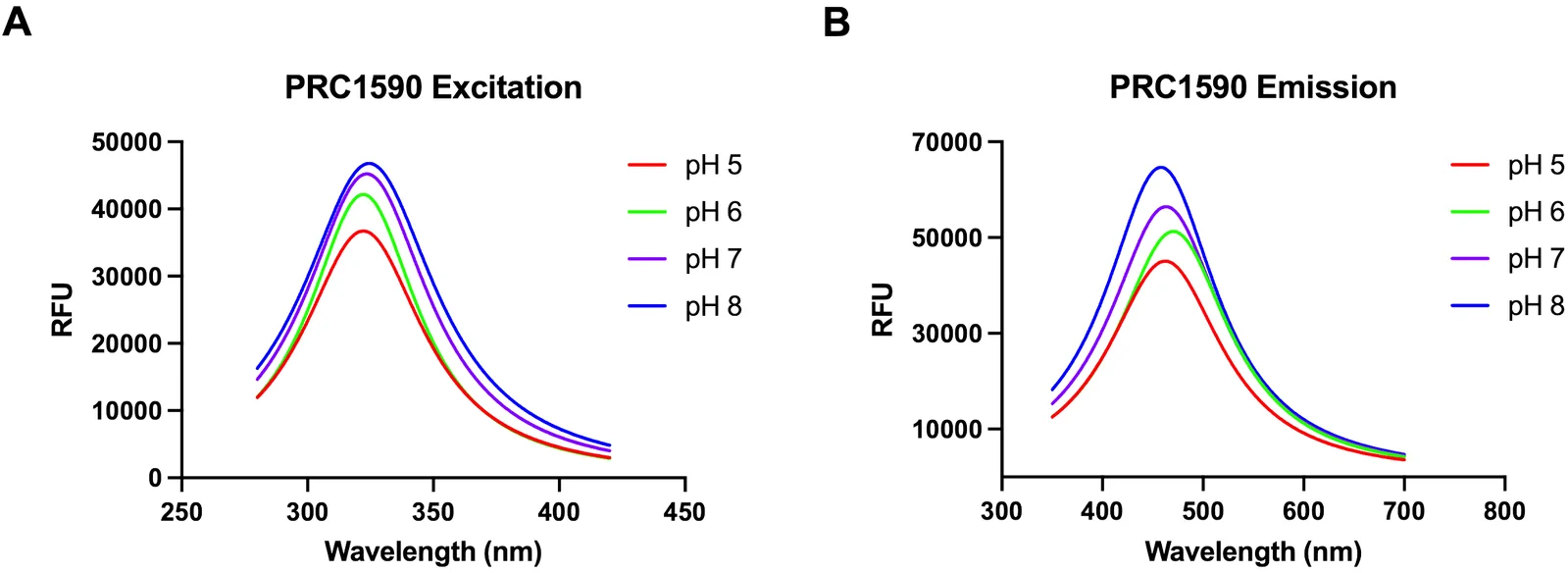
Excitation and emission spectra for PRC1590 at 1 μM and varying pHs. **(A)** Excitation spectra with a fixed emission of 470 ± 20 nm. **(B)** Emission spectra with a fixed excitation of 350 ± 20 nm. Additional spectra are reported in **Supplementary Table 2**.

**Figure 2:**
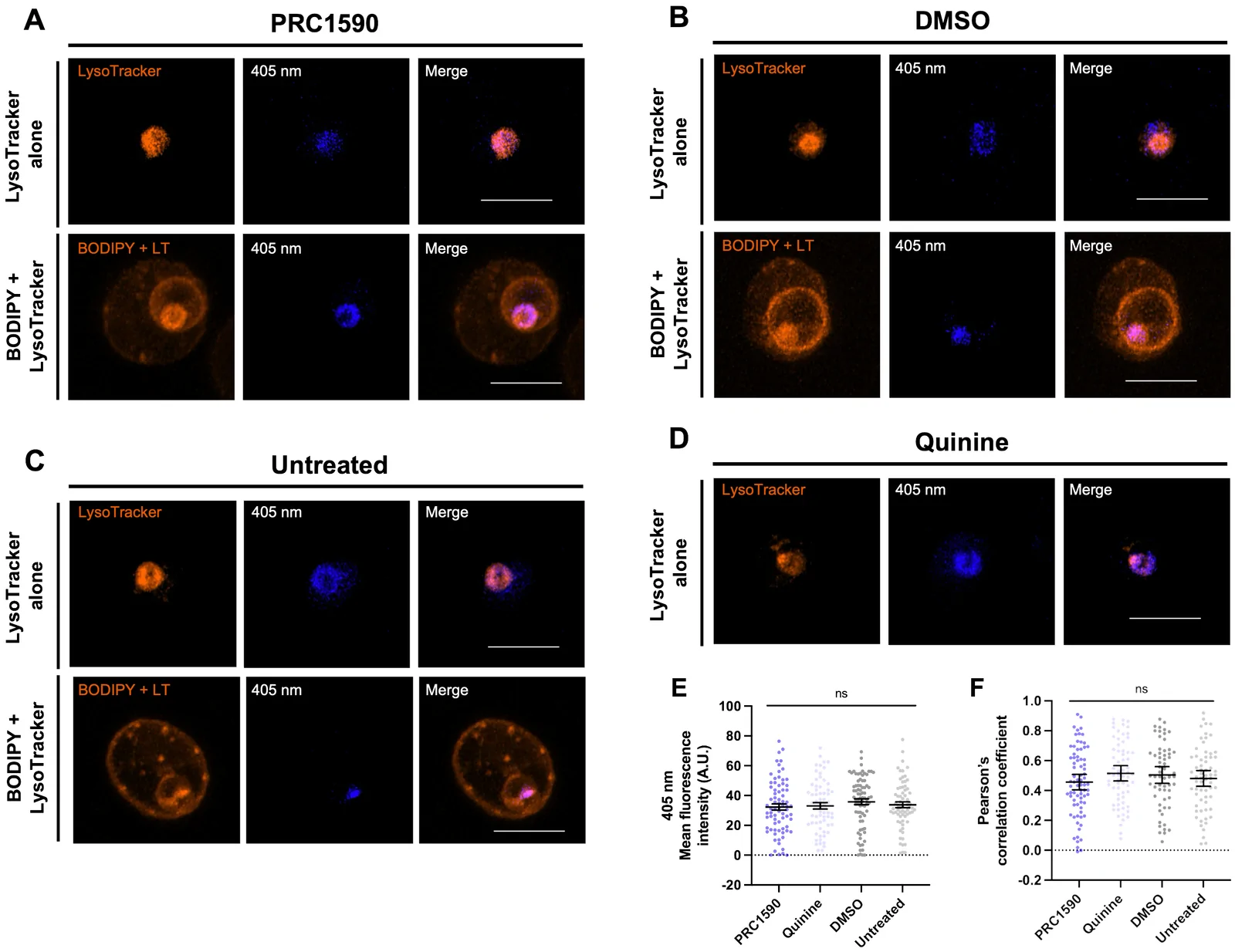
Microscopy co-localization experiments were conducted in parallel for **(A)** 1 μM PRC1590, **(B)** DMSO, **(C)** untreated parasites, and **(D)** parasites treated with 1 μM quinine (ex/em: 405 nm/460 ± 20 nm). LysoTracker (LT) and BODIPY were used to image the DV and membranes of the malaria parasites, respectively (ex/em: 561 nm/600 ± 30 nm). Images were obtained using the same microscopy settings, and 60 images were acquired for each condition. Scale bar = 5 μm. **(E)** Scatterplot representing mean fluorescence intensity of signal in 405 nm channel and **(F)** Pearson’s correlation coefficient between 561 nm (LysoTracker) and 405 nm for each condition. Error bars represent the standard error of the mean (SEM). Statistical significances for E and F were determined using one-way ANOVA test. ns = not significant. There was no significant statistical or practical difference in mean fluorescence intensity (*p* = 0.627, Cohen's *f* = 0.078) nor Pearson’s correlation coefficients (*p* = 0.387, Cohen's *f* = 0.108) between conditions. Additional images and statistical analysis are reported in the **Supplementary Materials** (**Supplementary Figures 1-4**; **Supplementary Tables 3-6**).

**Figure 3:**
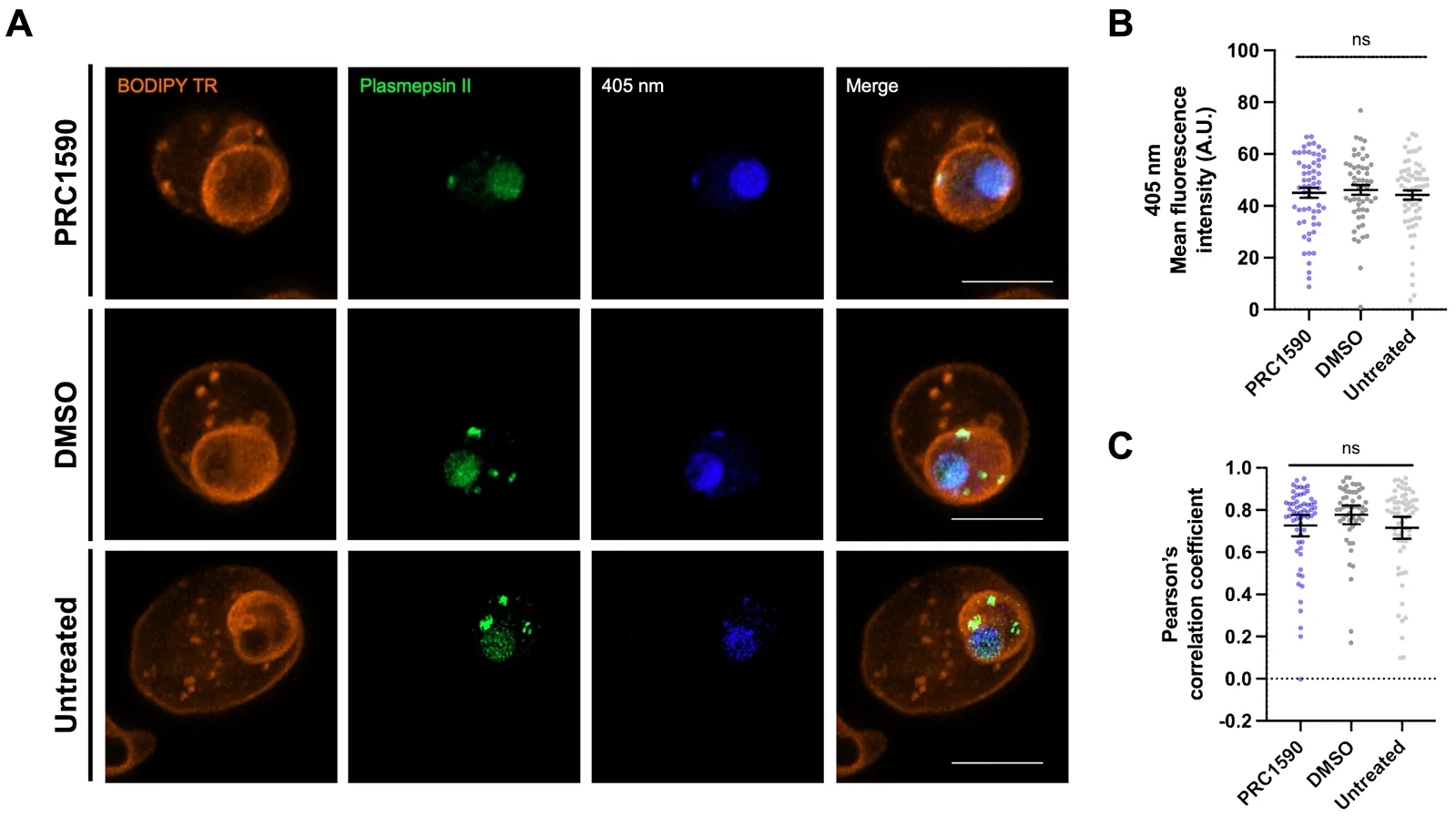
**(A)** Representative images of Plasmepsin II-GFP (proPM II-GFP) parasites treated with 1 μM PRC1590, DMSO, or left untreated. proPM II-GFP (ex/em: 488 nm/525 ± 25 nm) localizes to the digestive vacuole and BODIPY TR is a membrane stain (ex/em: 561 nm/600 ± 30 nm), allowing for visualization of the malaria parasite. Scale bar = 5 μm. **(B)** Scatterplot showing mean fluorescence intensity for each condition. Error bars represent the standard error of the mean (SEM). **(C)** Scatterplot showing Pearson’s correlation coefficient between 488 nm (proPM II-GFP) and 405 nm for each condition. Error bars = SEM. Statistical significances for B and C were determined using one-way ANOVA tests. ns = not significant. There was no significant statistical or practical difference in mean fluorescence intensity (*p* = 0.311, Cohen's *f* = 0.263) nor Pearson’s correlation coefficients (*p* = 0.196, Cohen's *f* = 0.138) between conditions. Additional images and statistical analysis are reported in the **Supplementary Materials** (**Supplementary Figures 5-7**; **Supplementary Tables 7-10**).

In this work, we aimed to assess the subcellular localization of a previously reported tetrahydro-β-carboline antimalarial compound, PRC1590, using its fluorescent properties (**Figure 1**) (Almolhim et al., 2022). PRC1590 fluoresces under UV illumination, and structurally related compounds have been localized in *P. falciparum* using their intrinsic fluorescence (Arzel et al., 2001; Korkor et al., 2020). Using previously described imaging methods, we observed fluorescent signal when parasites were treated with PRC1590. However, we observed comparable mean fluorescence intensity when imaging both DMSO-treated and untreated control parasites (**Figures 2**, **3**), indicating that the apparent localization of PRC1590 represents a false positive finding.

**Figure 4:**
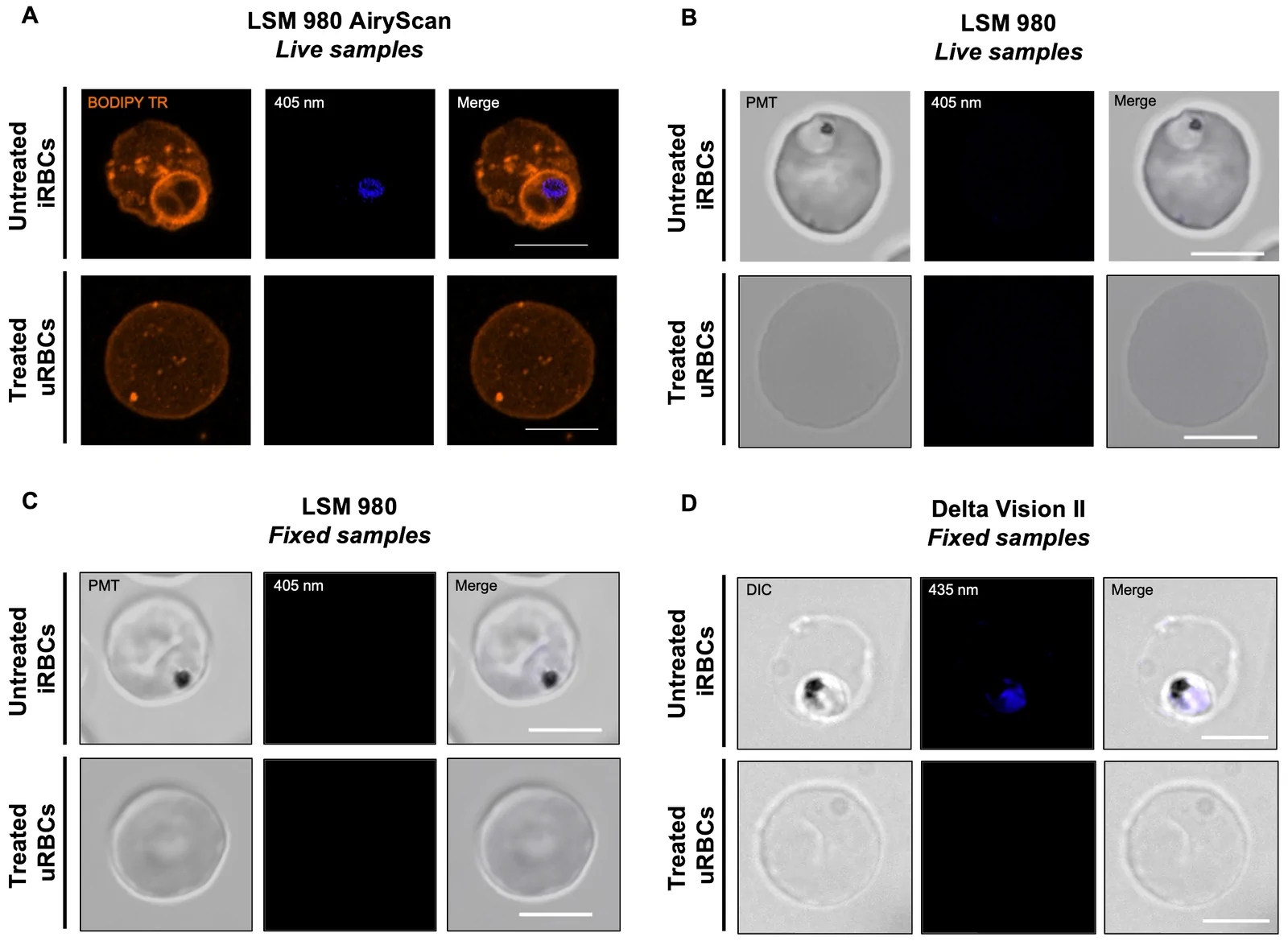
Autofluorescence of untreated infected red blood cells (iRBCs) and uninfected red blood cells (uRBCs) treated with 1 μM PRC1590. Live images of untreated iRBCs and treated uRBCs were obtained with **(A)** LMS 980 with AiryScan 2 and **(B)** LSM 980 confocal microscope. Fixed images of untreated iRBCs and treated uRBCs without stain with **(C)** LSM 980 confocal microscope and **(D)** Delta Vision II microscope. Scale bar = 5 μm. Additional images are shown in **Supplementary Figure 8**.

Previously published studies that have localized intrinsically fluorescent antiparasitic compounds often omit the reporting of negative controls. Therefore, it is possible that localization results for these compounds may be inaccurately represented in the literature when negative controls are not included in the experimental design. Some studies have imaged a compound with multiple stains (Purfield et al., 2009; Bouquet et al., 2012; Yang et al., 2016; Korkor et al., 2020), which may provide more compelling evidence of a compound’s localization but does not substitute a negative control. Other studies have reported images of treated parasites at varying drug incubation times (Boibessot et al., 2002; Mathis et al., 2006; Wilson et al., 2008; Giordani et al., 2014; Racané et al., 2023), varying drug concentrations (Yang et al., 2016), or treated parasites without the addition of subcellular makers (Arzel et al., 2001; Korkor et al., 2020) to demonstrate drug accumulation and localization. Based on available literature, it appears that only one study has documented images of drug-treated uninfected red blood cells as a negative control (Korkor et al., 2020). However, utilizing uninfected red blood cells as negative controls for imaging is not suitable due to autofluorescence of the malaria parasite (Garrido-Tamayo et al., 2025) (**Figure 4**). Thus, based on our attempts to troubleshoot imaging intrinsically fluorescence antimalarials, we have identified several critical methodological considerations that may assist other researchers interested in performing imaging studies to assess the subcellular localization of intrinsically fluorescent small molecules.

## 2 Results

### 2.1 False positive findings when imaging intrinsically fluorescent compounds

We hypothesized that PRC1590 has a mechanism of action related to the digestive vacuole (DV) of the malaria parasite because this compound has peak potency in the trophozoite stage and its mechanism of resistance is mediated by the DV membrane transporter, PfMDR1 (Bremers et al., 2025). We used quinine as a positive control because previous work utilized quinine’s intrinsic fluorescence and identified co-localization with the DV stain LysoTracker Red-DND 99 (Bohórquez et al., 2012). We were able to replicate the reported localization findings for quinine (**Figure 2D**); however, untreated and DMSO-treated parasites stained with LysoTracker produced similar mean fluorescence intensity at 405 nm as parasites treated with PRC1590 or quinine (**Figure 2E**). We identified that there was neither statistical significance (*p* = 0.627) nor practical significance (Cohen's *f* = 0.078) between the mean fluorescence intensity for the four conditions with a one-way ANOVA test (**Supplementary Tables 3-6**). These findings indicate that the distribution of mean fluorescence intensity largely overlaps for treated and untreated parasites (**Supplementary Tables 3, 4**). Additionally, there was no significant difference between Pearson’s correlation coefficients between conditions (*p* = 0.387, Cohen's *f*= 0.108).

We then explored co-localization of PRC1590 with the endogenous DV marker Plasmepsin II that has previously been tagged with GFP (termed proPM II-GFP) (Klemba et al., 2004). Protein fluorophores often perform better than stains due to their improved molecular brightness (Grimm and Lavis, 2022). Again, untreated controls showed fluorescence with a mean intensity similar to that observed after PRC1590 treatment at 405 nm (**Figure 3**). These imaging data show no statistically significant differences between mean fluorescence intensity (*p* = 0.311, Cohen's *f* = 0.263) nor Pearson’s correlation coefficient (*p* = 0.196, Cohen's *f* = 0.138) for treated and untreated parasites (**Supplementary Tables 7-10**). Thus, localization of PRC1590 to the DV using its intrinsic fluorescence could not be confirmed under the tested conditions.

### 2.2 Spectral overlap and autofluorescence contribute to imaging artifacts

Based on the Pearson’s correlation coefficients for both LysoTracker and proPM II-GFP to the 405 nm channel (**Figures 2E, C**), we suspect that spectral overlap contributes to the false positive localization findings. Spectral overlap, or fluorescence emission crosstalk, occurs when the excitation/emission spectra of a fluorophore overlap with another fluorophore. This overlap causes fluorescent signal to bleed through into the other channel. We observed that the proPM II-GFP parasites (488 nm channel) had a greater Pearson’s correlation coefficient with the 405 nm channel than LysoTracker-stained parasites (**Figures 2F, C**). Our data suggest that spectral overlap contributed to the false positive localization findings of PRC1590 since the 488 nm channel for visualizing proPM II-GFP is closer to the 405 nm channel used to image PRC1590 when compared to LysoTracker (561 nm channel). Additionally, we identified no statistically significant difference between the mean Pearson’s correlation coefficient across PRC1590, DMSO, and untreated conditions (**Figures 2F, C**; **Supplementary Tables 5**, **9**). These data suggest that spectral overlap can produce false positive co-localization results when imaging intrinsically fluorescent small molecules. Experiments that aim to image intrinsically fluorescent compounds should consider stains that have a greater separation in wavelengths to prevent false positive findings.

To assess if autofluorescence of the malaria parasite also contributed to the imaging artifacts we observed, we imaged parasites with different microscopes under live and fixed conditions. We detected cell autofluorescence for two imaging conditions (**Figures 4A–D**). We observed autofluorescence when imaging live parasites incubated with the membrane stain BODIPY using confocal microscopy (**Figure 4A**). Fixed parasites imaged with Delta Vision II also showed autofluorescence (**Figure 4D**). Delta Vision II uses widefield, meaning the entire sample is illuminated at a specific wavelength, which can increase fluorescence background and visualization of autofluorescence (Stephens and Allan, 2003). Thus, autofluorescence is a prevalent issue that requires specific matched negative controls.

## 3 Discussion

### 3.1 Experimental considerations when performing imaging studies with small molecules to assess subcellular localization

In summary, our results suggest that efforts to localize two antimalarial compounds in *P. falciparum* can produce false-positive findings due to spectral overlap and autofluorescence (**Figures 2**-**4**). These observations underscore the value of including additional, well-matched controls when studying intrinsically fluorescent compounds in the malaria parasite. We recognize that this work reflects imaging data from a limited number of compounds and imaging settings in a single *Plasmodium* species. With these limitations in mind, we hope these findings will be useful to fellow parasitologists by highlighting potential challenges associated with imaging intrinsically fluorescent molecules. Future studies may build on this work by examining a broader range of structurally diverse compounds in other parasitic species, as well as by further evaluating instrument-specific crosstalk. We therefore offer the following considerations for researchers interested in assessing the localization of antiparasitic compounds.

#### 3.1.1 Assess the excitation/emission spectra of a compound at a relevant concentration and various pHs

Relying on spectral scanning during microscopy to identify peak excitation and emission of a small molecule can be misleading. A more informative approach is to assess the excitation and emission spectra for the compound of interest at a concentration relevant for imaging before conducting microscopy experiments. Additional biologically relevant factors should be considered when assessing the spectra of an intrinsically fluorescent compound such as pH. Excitation and emission can be affected by pH (Grimm and Lavis, 2022), therefore, intrinsically fluorescent compounds could preferentially co-localize with acidic organelles. Measuring excitation and emission at varying pHs as shown in **Figure 1** could help address this concern. Biological contexts can also cause shifts in excitation/emission, molecular brightness, and the fluorescence lifetime of a molecule (Grimm and Lavis, 2022). For instance, metabolization or modification of the drug can potentially impact its signal. Thoughtful negative controls can aid in interpretation of imaging data if biological context alters the intrinsic fluorescence of a compound.

#### 3.1.2 Proper negative controls can aid interpretation of localization data

Autofluorescence of a biological sample can produce imaging artifacts. Aromatic proteins, coenzymes like NADPH, lipids, and lipopigments are autofluorescent (Monici, 2005), meaning these molecules act as endogenous fluorophores when excited by suitable wavelengths. For example, researchers have used the autofluorescent properties of the malaria parasite to identify infection in red blood cells (Garrido-Tamayo et al., 2025). Their findings indicate that parasite autofluorescence at approximately 320 nm wavelength serves as a predictive marker for infection (Garrido-Tamayo et al., 2025). In our literature search, We found only one study imaging intrinsically fluorescent antiparasitic molecules that used uninfected cells as negative controls (Korkor et al., 2020). However, in our study, we did not observe a clear localization of PRC1590 within uninfected red blood cells across different microscopes and conditions, but we did observe an imaging artifact in untreated parasites using two different microscopes (**Figure 4**). Because we observed autofluorescence when imaging the malaria parasite, using untreated and vehicle-treated parasites as negative controls while maintaining the same imaging parameters across conditions are recommended.

#### 3.1.3 Subcellular accumulation is not sufficient evidence of target engagement

We would like to underscore a few final considerations when imaging an intrinsically fluorescent molecule. While utilizing the intrinsic fluorescence of a small molecular is attractive, there are a number of technical challenges that we outlined in this work. Addition of a fluorophore such as coumarin or 7-Nitrobenz-2-oxa-1,3-diazole (NBD) may be more useful for imaging by increasing the drug’s molecular brightness (McQueen et al., 2017; Attram et al., 2023). However, researchers may wish to carefully consider the value of localizing a small molecule in the parasite of interest. Apparent subcellular accumulation alone may not provide sufficient evidence of target engagement, particularly for compounds whose fluorescence and physicochemical behavior can be influenced by the cellular context. We therefore encourage researchers to interpret compound accumulation with care, as its localization may not necessarily reflect the compound’s mechanism of action. For instance, new permeability pathways (NPPs) of malaria-infected RBCs impact the uptake of molecules such as vitamin B5 (Ginsburg et al., 1983; Saliba et al., 1998), meaning drugs could readily accumulate in the parasite but not necessarily at the location of their target. Because the permeability of the host erythrocyte is modified by NPPs, uRBCs are not suitable negative controls for co-localization experiments in the malaria parasite. Additionally, certain physiochemical properties impact accumulation of a drug into cellular compartments like the DV. Weak bases like chloroquine accumulate in the DV due to the lysomotrophic effect (Krogstad and Schlesinger, 1986; Ch’Ng et al., 2011). These compounds are able to pass through the DV membrane due to their lipophilicity, and subsequently get protonated and trapped in this acidic organelle (Ch’Ng et al., 2011). Even with reliable localization findings, drug accumulation in the malaria parasite has the potential to complicate interpretation of the MOA of a compound.

Researchers may also wish to consider complementary approaches, beyond localization studies, to assess a drug’s MOA. In the context of malaria, comparing digestive vacuole phenotypes between untreated and treated parasites may provide helpful insight into whether a compound contributes to DV disruption (Edgar et al., 2021). Additionally, previous work in antiparasitic drug discovery have imaged parasites with different subcellular markers following drug treatment, an approach that may be more informative than assessing compound accumulation alone (Dembele et al., 2017; Thomas et al., 2018). While these alternative qualitative approaches can serve as useful starting point for target identification, other methods such as resistance selection when feasible using *in vitro* evolution and whole genome sequencing analysis (IVEWGA), protein stability assays, or chemoproteomic approaches may provide a better understanding of a small molecule’s MOA.

## 4 Methods

### 4.1 LysoTracker co-localization experiments

Methods pertaining to chemicals, *in vitro P. falciparum* cultures, and spectral analysis are detailed in the **Supplementary Materials**. Across all experiments, parasites were synchronized with one round of 5% sorbitol (Sigma-Aldrich, St. Louis, MO, USA) treatment during the ring stage. Once in the trophozoite stage, cultures at 5% hematocrit and a parasitemia greater than 7% were incubated with 1 μM of LysoTracker Red-DND 99 (Thermo Fisher Scientific, Waltham, MA, USA) for 1 hour at 37 °C in reduced oxygen conditions, protected from light. Following incubation, cultures were washed once with pre-warmed complete RPMI media and resuspended in RPMI. Cultures were then incubated for 30 minutes with 1 μM of drug (Quinine or PRC1590), or equivalent volume of DMSO without further washing. Untreated parasites were prepared similarly except no drug or drug vehicle were added to the culture during this final incubation step.

For imaging, 35 mm glass dishes and the Zeiss LSM 980 Confocal Microscope with Airyscan 2 were used. The 35 mm glass dishes (Cellvis, Mountain View, CA, USA) were coated with poly-L-lysine (Sigma-Aldrich, St. Louis, MO, USA) for 1 hour at 37 °C and then washed twice with MilliQ water. Parasites were diluted in 1xPBS, then transferred to dishes. Images were acquired using the Zeiss LSM 980 confocal system with an inverted Axio Observer 7 microscope stand that uses laser (405–730 nm) illumination. We used a 100x/1.4 NA Plan Apochromat oil immersion objective with Airyscan 2 for improved resolution by a factor of 1.7. Fluorescence was captured sequentially with a 405 nm diode laser at 3% excitation power, and a 561 nm DPSS laser at 2% power. The final image pixel size was 0.04 μm/pixel. Additional microscope specifications and acquisition parameters can be found in the **Supplementary Table 1**.

We used *a priori* power analysis to determine the sample size before imaging using G*Power (Release 3.1.9.6) (Faul et al., 2007). Based on this analysis, we set out to image 60 fields per condition for experiments we planned to quantify and statistically compare. Thus, we captured 60 images across six independent biological replicates for the LysoTracker only experiments. We imaged ten fields for each condition per biological replicate. Across all experiments, we randomly selected imaging fields and then centered a single parasite in the field using brightfield. While we rarely excluded fields, we did not image fields that were overcrowded or contained debris. All imaged fields were included in the analysis. Ten to 12 z-stacks (z-step: 0.19 nm) were collected per image depending on the maturity of the trophozoite. Images were captured by Zen Blue 3.3 software, processed with Airyscan 2, and then displayed as a maximum intensity projection.

To quantify fluorescence intensity, the measure function in ImageJ was used as previously described (Shihan et al., 2021). Mean fluorescence intensity was calculated by using the Freehand Selection tool to precisely select and then measure the fluorescence intensity for regions of interest in the 405 nm channel from raw, uncropped images. Measurements for each image (n= 60 images per condition) were taken three times and averaged. A one-way ANOVA test was used to statistically compare the mean fluorescence intensities between the four conditions using GraphPad Prism (Version 11.0.2). We then used a previously described CellProfiler (Version 4.2.5) workflow (Stirling et al., 2021) to calculate the Pearson’s correlation coefficients between the 405 nm and 561 nm channels. First, illumination variation was corrected, images were then aligned, and the Pearson’s correlation coefficient was measured for the 60 raw images. Again, we conducted a one-way ANOVA test to statistically compare the correlation coefficients between the four conditions. We report the full statistical analysis in the **Supplementary Materials** (**Supplementary Tables 3**-**10**).

For imaging experiments with BODIPY and LysoTracker, 1 μM of BIODIPY TR ceramide (Thermo Fisher Scientific) was added at the same time as LysoTracker and incubated for 1 hour at 37 °C under reduced oxygen conditions while protected from light. These cultures were washed twice with warm RPMI then resuspended in RPMI. Cultures were then incubated with 1 μM of PRC1590, the equivalent volume of DMSO, or were not treated for 30 minutes. Thirty images across three independent biological replicates were captured with the same microscope specifications and acquisition settings as the LysoTracker only experiments. These images were not used to quantify mean fluorescence intensity nor to determine Pearson’s correlation coefficients.

### 4.2 Plasmepsin II-GFP co-localization experiments

We used the previously generated Plasmepsin II GFP-tagged (proPM II-GFP) chimeric strain with a 3D7 background (Klemba et al., 2004), which was kindly provided by Dan Goldberg (Washington University, St. Louis, USA). Synchronized trophozoite parasites with a parasitemia greater than 7% were stained with 1 μM BIODIPY TR ceramide and incubated in 6 well plates for 1 hour at 37 °C, under reduced oxygen conditions in hypoxic chambers. Following incubation, parasites were washed three times and resuspended with warm RPMI. PRC1590 at 1 μM or an equivalent volume of DMSO were then added to the resuspended pellet and incubated for 30 minutes at 37 °C with no wash step prior to imaging. Untreated parasites also underwent the same incubation conditions and were then imaged. All microscope and acquisition settings were maintained between conditions.

We imaged these proPM II-GFP with the Zeiss LSM 980 Confocal Microscope with Airyscan 2 and 35 mm glass dishes. The microscope settings were optimized to capture fluorescence using: 1) a 561 nm DPSS laser at 2% excitation power, 2) a 405 nm diode laser at 3% power, and 3) a 488 nm diode laser at 2% excitation power. Z-stack images were collected, processed with Airyscan, then images were displayed with maximum intensity projection (**Supplementary Table 1**). We conducted four independent biological replicates and imaged 15 fields per replicate for each condition to capture 60 total images per condition. We centered a single trophozoite in an imaging field and included all fields in the analysis. Brightness and contrast adjustments were made using the Zen Blue 3.3 software for display purposes only. Similar to the LysoTracker experiments, mean fluorescence intensity was calculated using ImageJ (Shihan et al., 2021), and Pearson’s correlation coefficients for all conditions were determined with CellProfiler (Stirling et al., 2021), and statistical analyses were performed using GraphPad Prism.

### 4.3 Autofluorescence imaging experiments

Uninfected RBCs (uRBCs) and infected RBCs (iRBCs) were maintained at 5% hematocrit across conditions. We first imaged parasites using the LSM 980 microscope with Airyscan 2 processing at 100x objective. uRBCs and iRBCs were incubated with 1 μM of BIODIPY TR ceramide for 1 hour at 37 °C under reduced oxygen conditions, protected from light. These cultures were washed three times and then resuspended with warm RPMI. Following staining with BODIPY TR, we added 1 μM PRC1590 to uRBCs or no drug to iRBCs and incubated these cultures for 30 minutes at 37 °C under reduced oxygen conditions while protected from light. Imaging parameters were the same for drug-treated uRBCs and untreated iRBCs for all autofluorescence imaging experiments. Additionally, we used the same imaging parameters from the LysoTracker co-localization experiments to assess autofluorescence with Airyscan 2 processing (**Supplementary Table 1**). We captured 30 images across three independent biological replicates.

For confocal imaging with LSM 980, live or fixed parasites were added to poly-L-lysine coated dishes as described above. Both live and fixed parasites were imaged under the same conditions for confocal microscopy with LSM 980: the T-PMT filter was illuminated with the 561 nm laser at 1% excitation power to capture brightfield images, and the 405 nm laser was used at 3% power to capture any autofluorescence. Cultures were visualized with the 100x objective, three to five z-stacks were taken per image and deconvolved to display their maximum projection. Thirty images were taken for both live and fixed conditions across three independent biological replicates. Parasites were fixed using a 2x fixative solution that consisted of 8% paraformaldehyde (Electron Microscopy Sciences, Hatfield, PA, USA), 0.03% glutaraldehyde (Electron Microscopy Sciences), in a final volume of 1xPBS. One mL of 5% hematocrit cultures (for both iRBC and uRBC conditions) were spun down, washed in 1xPBS, and resuspended in 200 μL of 1xPBS. Two hundred μL of the 2x fixative solution was added and incubated for 30 minutes at room temperature while protected from light. After fixing, the pellet was washed with 1xPBS. This washed pellet was then incubated with 0.1 mg/mL of NaBH_4_ for 10 minutes, rocking at room temperature, to remove free aldehydes. Following NaBH_4_ incubation, the pellet was washed with 1xPBS and was ready for imaging.

Lastly, we performed autofluorescence experiments using the Delta Vision II microscope system with an inverted Olympus IX-71 microscope. Fixed parasites were applied to poly-L-lysine–coated glass coverslips and allowed to settle and adhere for 30 minutes; unadhered cells were then aspirated. The coverslip was then dipped in 1xPBS to remove any unbounded cells. Mounting media (25 mM Tris, 50% glycerol) was added to slides, followed by the coverslips being placed and then sealed using nail polish to prevent drying. The Pol filter was set to 50% power with 0.150 s exposure time to capture DIC images, and the DAPI (435 nm) filter was set to 32% power with a 1.500 s exposure to capture any autofluorescence. Thirty images were taken for iRBC and uRBC conditions across three biological replicates. Images were captured as a z-stack with CoolSnap HQ2 and a cooled CCD camera, deconvolved using SoftWorx, and displayed as a maximum intensity projection. Additional microscope specifications and acquisition parameters are reported in **Supplementary Table 1**.

## Data Availability

The original contributions presented in the study are included in the article/**Supplementary Material**. Further inquiries can be directed to the corresponding authors.
