# Supplementary material for: Navigating imaging artifacts: challenges with visualizing intrinsically fluorescent antimalarials
Source: Front Parasitol. 2026 Aug 17;5:1919904. doi: 10.3389/fpara.2026.1919904 (PMC13526941; doi:10.3389/fpara.2026.1919904)
Supplement: Supplementary file 1 [file SupplementaryFile1.pdf]

Supplementary Material

Navigating imaging artifacts: challenges with visualizing intrinsically fluorescent antimalarials

Emily K. Bremers[1,2], David Anaguano[2,3#], Paul R. Carlier[4,5], Vasant Muralidharan[2,3*], and Maria Belen Cassera[1,2*]
[1]Department of Biochemistry and Molecular Biology, University of Georgia, Athens, GA, USA
[2]Center for Tropical and Emerging Global Diseases, University of Georgia, Athens, GA, USA
[3]Department of Cellular Biology, University of Georgia, Athens, GA, USA
[4]Department of Pharmaceutical Sciences, University of Illinois Chicago, Chicago, IL, USA
[5]Department of Chemistry, University of Illinois Chicago, Chicago, IL 60612
[#]Current address: Gulbenkian Institute for Molecular Medicine, Lisbon, Portugal
*****Correspondence: Maria Belen Cassera maria.cassera@uga.edu and Vasant Muralidharan vasant@uga.edu

1 Extended Methods

1.1 Chemicals

PRC1590 (compound (*R*)-**3b** in reference 1) was previously synthesized and purified as described (1). Quinine was obtained from Sigma-Aldrich (St. Louis, MO, USA). Both compounds were reconstituted in DMSO.

1.2 Excitation/emission spectrum analysis

To assess the fluorescence intensity of PRC1590 at varying pHs, we used a plate reader (BioTek Cytation 5), and Ringer's solution based on a previous protocol with some modifications (2). Modified Ringer's solutions of varying pHs were made with 122.5 mM NaCl, 5.4 mM KCl, 1.2 mM $CaCl_2$, 0.8 mM $MgCl_2$, 11 mM D-glucose. For solutions at pH 5 and 6, 10 mM MES was added to the solution. For solutions at pH 7 and 8, 10 mM HEPES was added. One hundred µL of each buffer with 1 µM of PRC1590 was added to a 96 well plate and tested in technical duplicate. A blank for each buffer solution was also assessed. Excitation was measured from 300 to 700 nm with a fixed emission at 600 nm. Emission was measured with a fixed excitation of 480 nm and measured from 300 to 700 nm. Quantification was done in GraphPad Prism (GraphPad Software, Inc).

1.3 *Plasmodium falciparum in vitro* culture

The *P. falciparum* strains 3D7 (MRA-102) was obtained from MR4 (ATCC, Manassas, VA, USA; BEI Resources, NIAID, NIH). The *P. falciparum* Plasmepsin II-GFP tagged line was kindly supplied by Dan Goldberg (Washington University, St. Louis, USA) (3). Cultures were maintained at 5% hematocrit in O positive or A positive red blood cells (Grifols Bio Supplies Inc., Vista, CA, USA) and RPMI 1640 media (Thermo Fisher Scientific, Waltham, MA, USA). RPMI containing 2 g/L glucose, 5.94 g/L HEPES, 2.3 g/L sodium bicarbonate, 5 g/L Albumax I, and 50 mg/L hypoxanthine, all obtained from Sigma-Aldrich (St. Louis, MO, USA). Media was supplemented with 20 mg/L of gentamicin (Thermo Fisher Scientific). Parasite cultures were kept at 37°C, shaking, in reduced oxygen conditions (5% CO_2, 5% O_2, and 90% N_2).

frontiers

Table S1: Microscope specifications and acquisition parameters

FIGURES		MICROSCOPE SPECIFICATIONS			ACQUISITION PARAMETERS							
**Figure**		**System**	**Mag. /NA**	**WD**	**Frame size**	**Pixel size**	**z-step**	**z-stack range**	**Detector**	**Laser/Filter (Em. range)**	**Ex. Power**	**Pixel dwell time/ Exposure time**
2	a-d	Zeiss LSM 980	100x/ 1.4 (oil)	0.17 mm	1024 px x 1024 px	0.04 µm/px	0.19 µm	10-12	Airyscan 2 SR	405 nm (420-480 nm)	3%	2 µs
										561 nm (570-630 nm)	2%	1 µs
3	a	Zeiss LSM 980	100x/ 1.4 (oil)	0.17 mm	1024 px x 1024 px	0.04 µm/px	0.19 µm	8	Airyscan 2 SR	405 nm (420-480 nm)	3%	2 µs
										488 nm (495-550 nm)	2%	1 µs
										561 nm (570-630 nm)	2%	1 µs
4	a	Zeiss LSM 980	100x/ 1.4 (oil)	0.17 mm	1024 px x 1024 px	0.04 µm/px	0.19 µm	10-12	Airyscan 2 SR	405 nm (420-480 nm)	3%	2 µs
										561 nm (570-630 nm)	2%	1 µs
	b-c	Zeiss LSM 980	100x/ 1.4 (oil)	0.17 mm	1024 px x 1024 px	0.08 µm/px	0.19 µm	3-5	GaAsP, T-PMT	405 nm (420-480 nm)	3%	2 µs
										T-PMT	Illuminated with 2% 561 nm diode laser	1 µs
	d	Delta Vision II	100x/ 1.4 (oil)	0.12 mm	1024 px x 1024 px	0.06 µm/px	0.5 µm	6-8	CCD	DAPI (409-456 nm)	32%	1.500 s
										Pol (DIC)	50%	0.150 s

Table Key: Mag.: Magnification, WD: Working distance, Em.: Emission, Ex.: Excitation

Table S2. Peak excitation and emission for PRC1590 and Quinine.

[Concentration] Compound	Peak excitation (nm)	Peak emission (nm)
[1 μM] PRC1590	325 ± 20 nm	460 ± 20 nm
[100 nM] Quinine	330 ± 20 nm	480 ± 20 nm

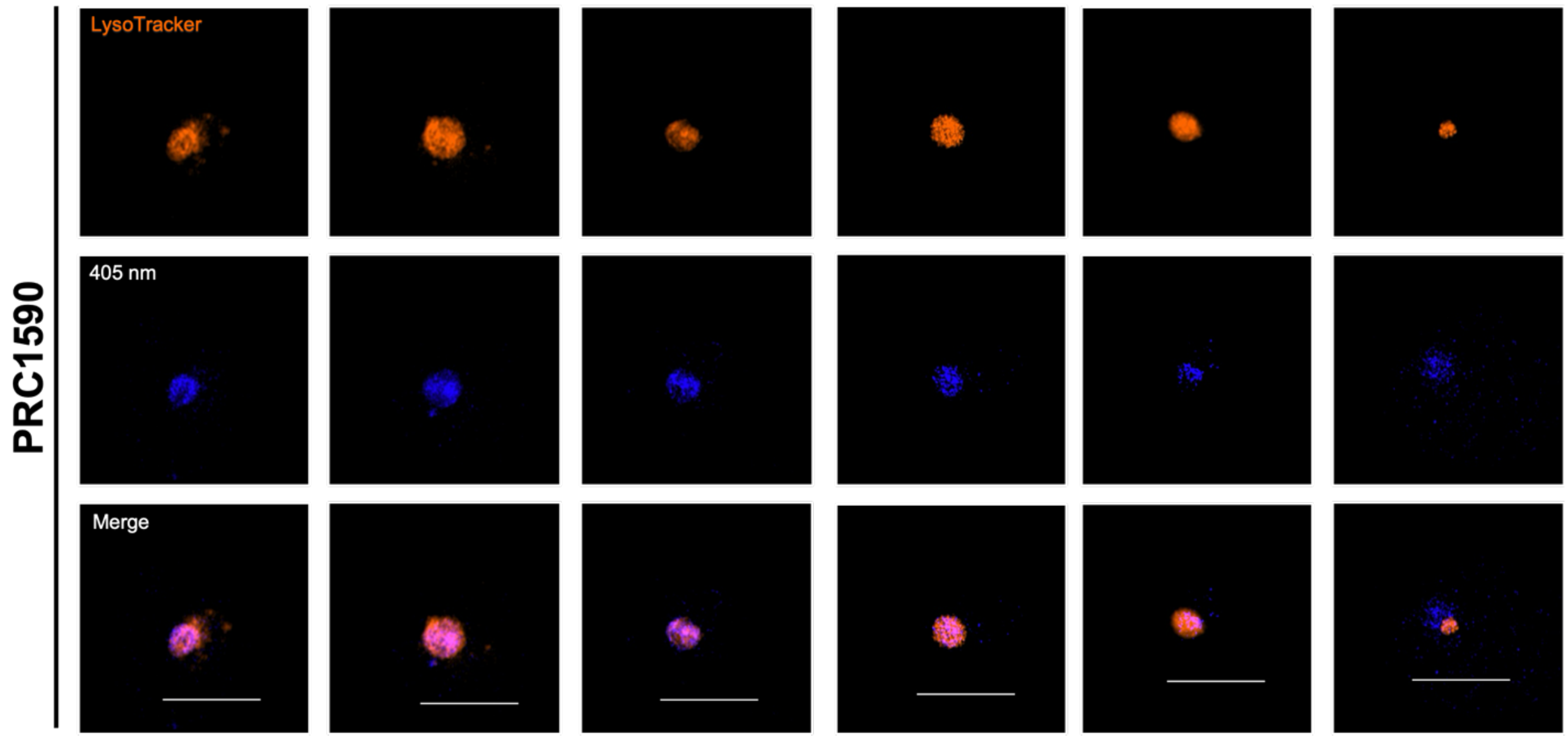

Figure S1. *Plasmodium falciparum* 3D7 strain stained with LysoTracker Red DND-99 and treated with 1 µM of PRC1590. Scale bar = 5 µm.

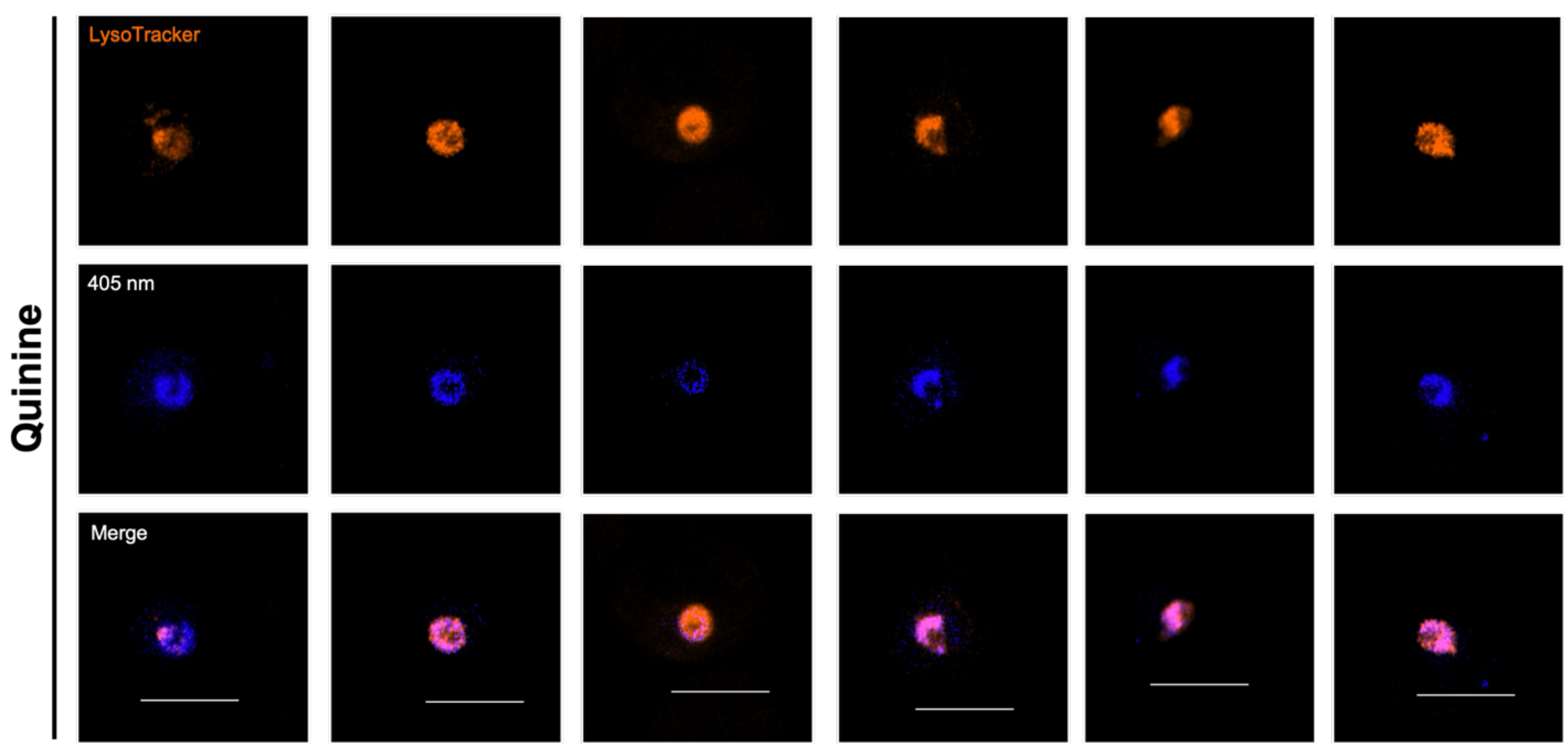

Figure S2. Imaging of *P. falciparum* 3D7 strain parasites stained with LysoTracker Red DND-99 and treated with 1 µM of Quinine. Scale bar = 5 µm.

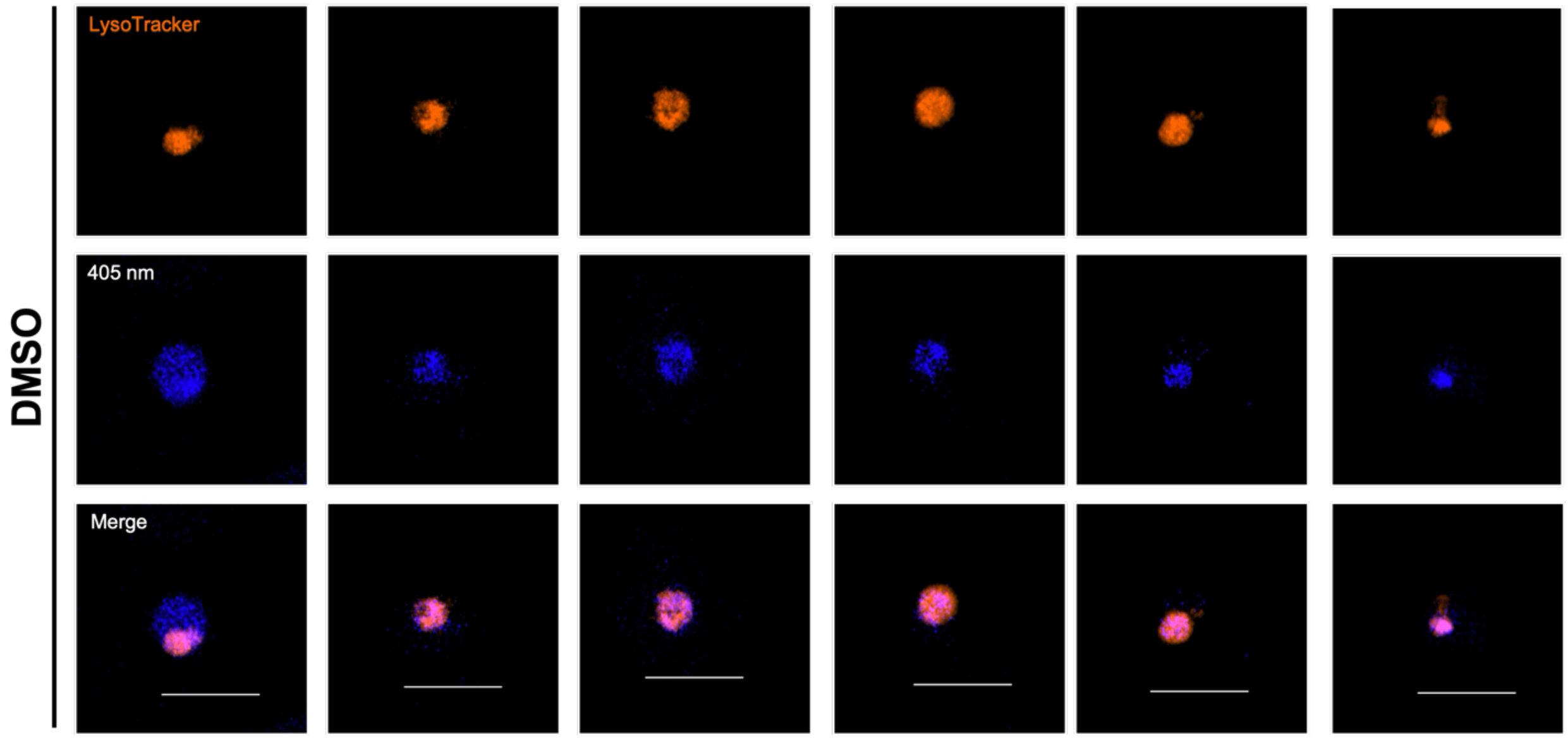

Figure S3. Imaging of *P. falciparum* 3D7 strain parasites stained with LysoTracker Red DND-99 and treated with equal volume of DMSO (0.1% DMSO). Scale bar = 5 µm.

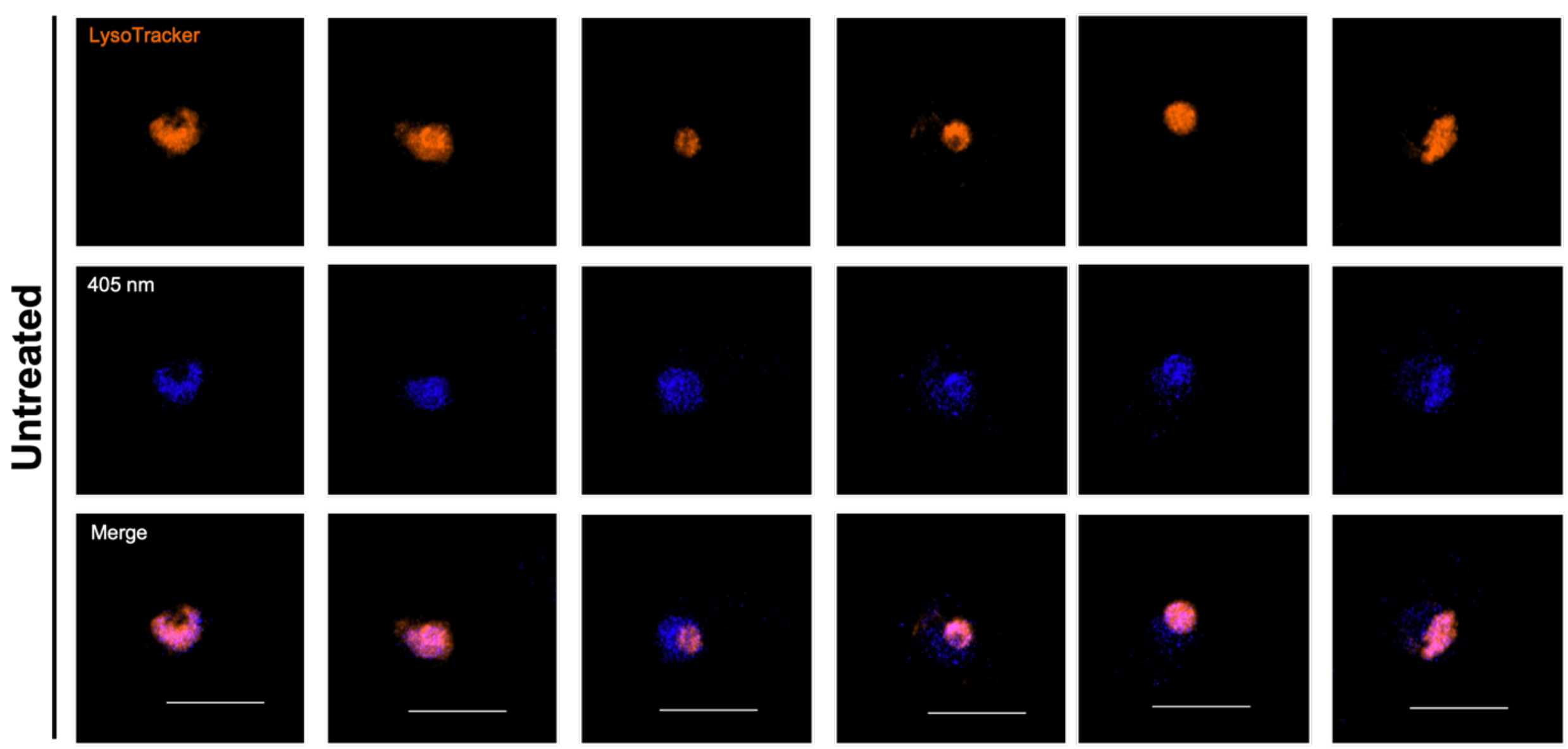

Figure S4. Imaging of untreated *P. falciparum* 3D7 strain parasites stained with LysoTracker Red DND-99. Scale bar = 5 µm.

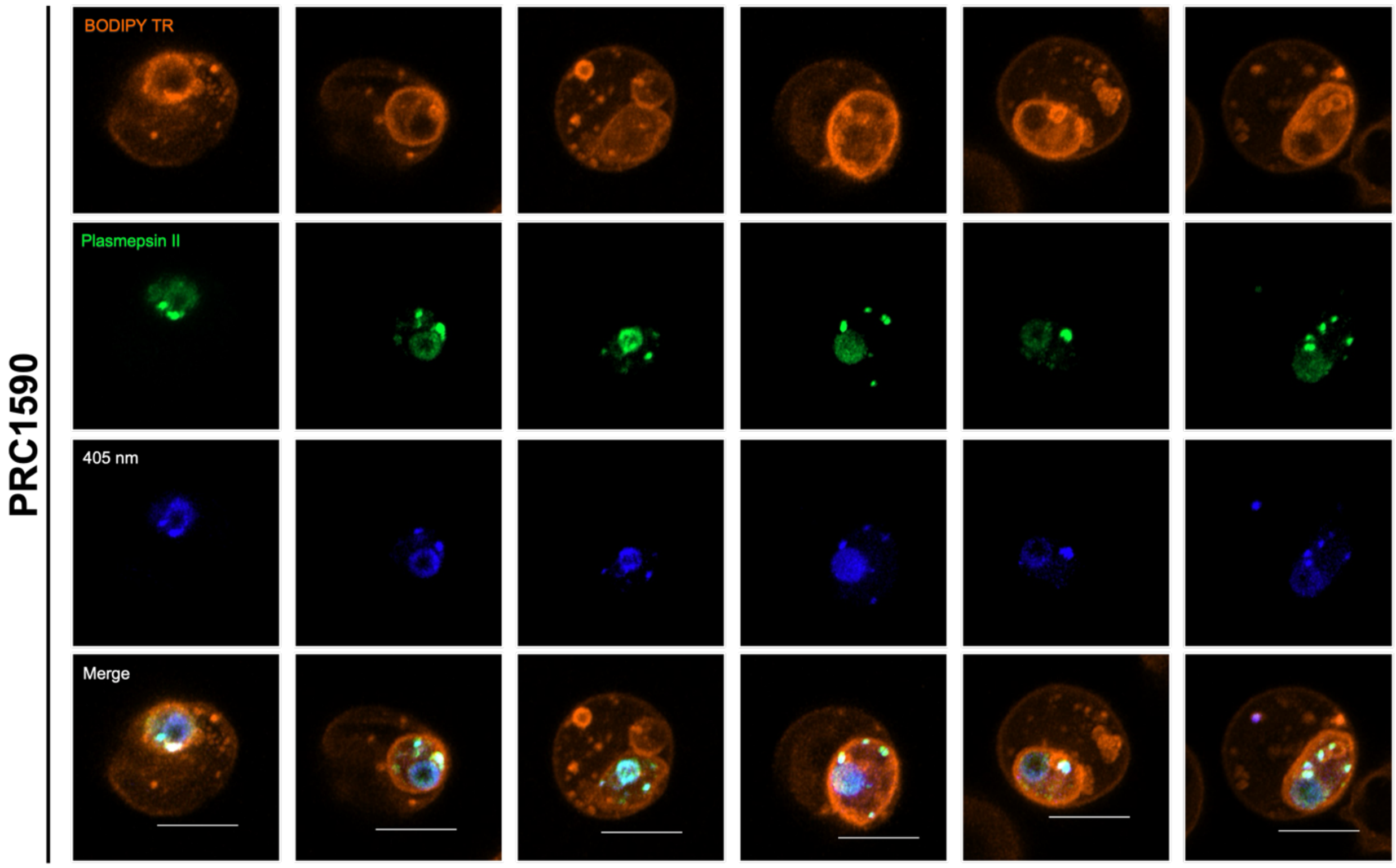

Figure S5. Imaging of *P. falciparum* Plasmepsin II-GFP parasites treated with 1 μM of PRC1590. Scale bar = 5 μm.

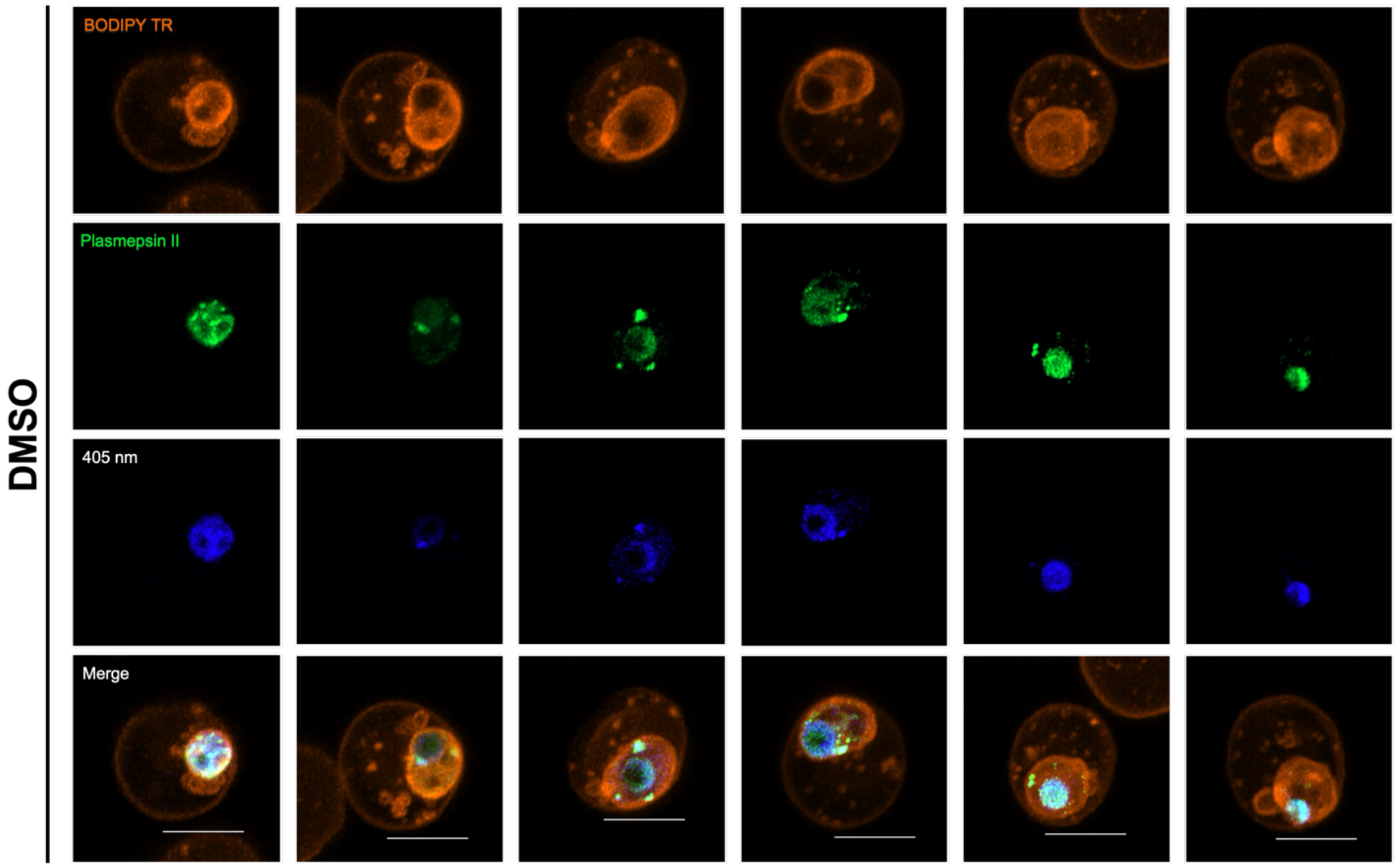

Figure S6. Imaging of *P. falciparum* Plasmepsin II-GFP parasites treated with equal volume of DMSO (0.1% DMSO). Scale bar = 5 μm.

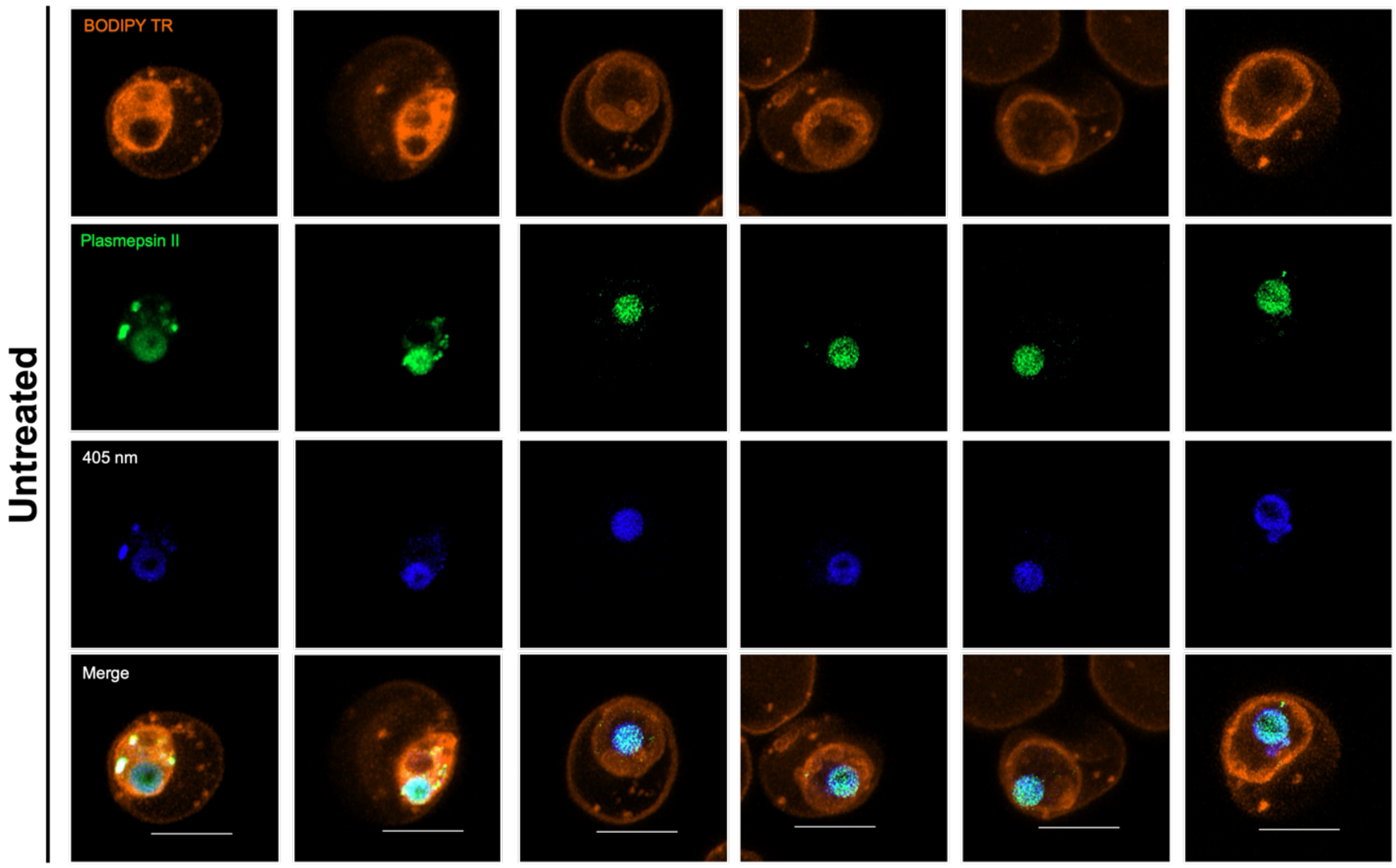

Figure S7. Imaging of untreated *P. falciparum* Plasmepsin II-GFP parasites under same imaging parameters. Scale bar = 5 µm.

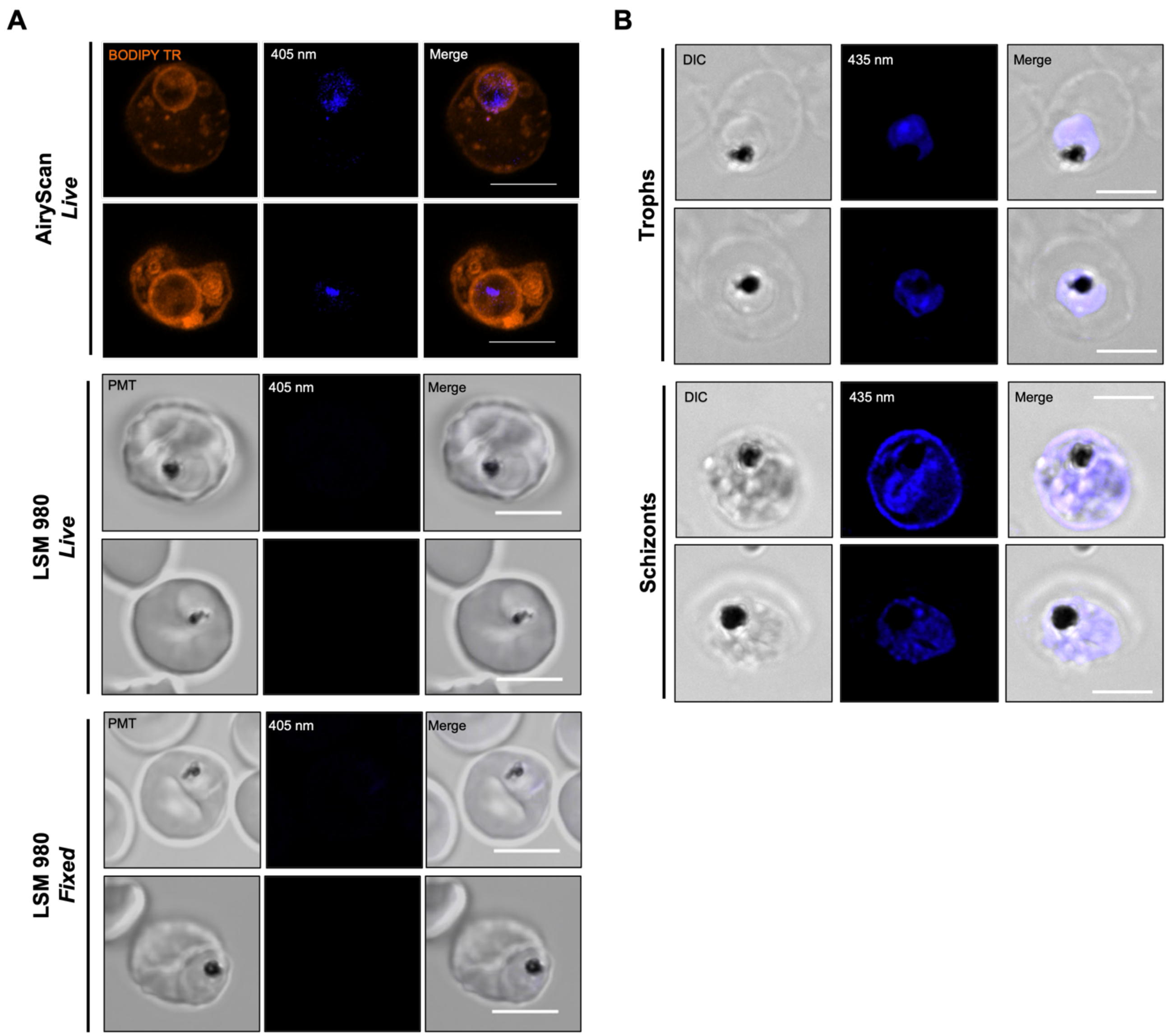

Figure S8. Autofluorescence of the malaria parasite. A) Additional images for experiments shown in Figure 4. Parasites are in the trophozoite stage B) Autofluorescence of fixed trophozoite and schizont stages of the malaria parasite when imaged with Delta Vision II. Scale bar = 5 µm.

Table S3: ANOVA summary statistics comparing the mean fluorescence intensity of channel 405 nm for the LysoTracker experiments.

**ANOVA summary**	
F	0.5822
P value	0.6271
P value summary	ns
η^2	0.006112
Partial η^2	0.006112
Cohen's f	0.07842

Table S4: Descriptive statistics comparing the mean fluorescence intensity of channel 405 nm across four conditions for the LysoTracker experiments.

	**PRC1590**	**Quinine**	**DMSO**	**Untreated**
Mean	32.28	33.09	35.78	33.77
Std. Deviation	18.00	17.35	17.35	16.04
Std. Error of Mean	2.078	2.169	1.940	1.931
Lower 95% CI	28.14	28.76	31.92	29.92
Upper 95% CI	36.42	37.42	39.64	37.63

Table S5: ANOVA summary statistics comparing the co-localization correlation coefficients for the LysoTracker experiments.

**ANOVA summary**	
F	1.014
P value	0.3869
P value summary	ns
η^2	0.01148
Partial η^2	0.01148
Cohen's f	0.1078

Table S6: Descriptive statistics comparing the co-localization correlation coefficients across four conditions for the LysoTracker experiments.

	**PRC1590**	**Quinine**	**DMSO**	**Untreated**
Mean	0.4559	0.5150	0.5034	0.4809
Std. Deviation	0.2264	0.2096	0.2168	0.2121
Std. Error of Mean	0.02597	0.02600	0.02800	0.02631
Lower 95% CI	0.4041	0.4630	0.4474	0.4284
Upper 95% CI	0.5076	0.5669	0.5594	0.5335

Table S7: ANOVA summary statistics comparing the mean fluorescence intensity of channel 405 nm for the Plasmepsin II-GFP experiments.

**ANOVA summary**	
F	0.3107
P value	0.7405
P value summary	ns
η^2	0.06458
Partial η^2	0.06458
Cohen's f	0.2628

Table S8: Descriptive statistics comparing the mean fluorescence intensity of channel 405 nm across three conditions for the Plasmepsin II-GFP experiments.

	**PRC1590**	**DMSO**	**Untreated**
Mean	44.75	47.00	45.74
Std. Deviation	3.591	4.202	4.280
Std. Error of Mean	1.796	2.101	2.140
Lower 95% CI	39.04	40.31	38.93
Upper 95% CI	50.47	53.69	52.55

Table S9: ANOVA summary statistics comparing the co-localization correlation coefficients for the Plasmepsin II-GFP experiments.

**ANOVA summary**	
F	1.646
P value	0.1957
P value summary	ns
η^2	0.01868
Partial η^2	0.01868
Cohen's f	0.1380

Table S10: Descriptive statistics comparing the co-localization correlation coefficients across three conditions for the Plasmepsin II-GFP experiments.

	**PRC1590**	**DMSO**	**Untreated**
Mean	0.7260	0.7771	0.7154
Std. Deviation	0.1970	0.1592	0.2094
Std. Error of Mean	0.02544	0.02208	0.02617
Lower 95% CI	0.6751	0.7328	0.6631
Upper 95% CI	0.7769	0.8215	0.7677

Supplemental References

1. Almolhim H, Ding S, Butler JH, Bremers EK, Butschek GJ, Slebodnick C, et al. Enantiopure Benzofuran-2-carboxamides of 1-Aryltetrahydro-β-carbolines Are Potent Antimalarials *In Vitro*. ACS Med Chem Lett. 2022 Feb 24;13(3):371–6. doi:10.1021/acsmedchemlett.1c00697

2. Reiling SJ, Rohrbach P. Uptake of a fluorescently tagged chloroquine analogue is reduced in CQ-resistant compared to CQ-sensitive Plasmodium falciparum parasites. Malar J. 2019 Dec;18(1):342. doi:10.1186/s12936-019-2980-y

3. Klemba M, Beatty W, Gluzman I, Goldberg DE. Trafficking of plasmepsin II to the food vacuole of the malaria parasite Plasmodium falciparum. J Cell Biol. 2004 Jan 5;164(1):47–56. doi:10.1083/jcb200307147
